# Patient and Clinician Perspectives on the Effectiveness of Current Telemedicine Approaches in Endocrinology Care for Type 2 Diabetes: Qualitative Study

**DOI:** 10.2196/60765

**Published:** 2025-03-11

**Authors:** Margaret Zupa, Megan Hamm, Lane Alexander, Ann-Marie Rosland

**Affiliations:** 1 Division of Endocrinology and Metabolism University of Pittsburgh School of Medicine Pittsburgh, PA United States; 2 Caring for Complex Chronic Conditions Research Center University of Pittsburgh School of Medicine Pittsburgh, PA United States; 3 Qualitative, Evaluation and Stakeholder Engagement Services Center for Research on Health Care Data Center University of Pittsburgh Pittsburgh, PA United States; 4 Division of General Internal Medicine University of Pittsburgh School of Medicine Pittsburgh, PA United States; 5 Center for Health Equity Research and Promotion Veterans Affairs Pittsburgh Health System Pittsburgh, PA United States

**Keywords:** diabetes, telemedicine, video visit, endocrinology, effectiveness, type 2 diabetes mellitus, patient, perspectives, qualitative interviews, clinicians

## Abstract

**Background:**

Since the rapid widespread uptake in 2020, the use of telemedicine to deliver diabetes specialty care has persisted. However, evidence evaluating patient and clinician perspectives on benefits, shortcomings, and approaches to improve telemedicine care for type 2 diabetes is limited.

**Objective:**

This study aims to assess clinician and patient perspectives on specific benefits and limitations of current telemedicine care delivery for type 2 diabetes and views on approaches to enhance telemedicine effectiveness for patients who rely on it.

**Methods:**

We conducted semistructured qualitative interviews with diabetes specialty clinicians and adults with type 2 diabetes. We used a qualitative description approach to characterize participant perspectives on care delivery for type 2 diabetes via telemedicine.

**Results:**

Both clinicians (n=15) and patients (n=13) identify significant benefits of telemedicine in overcoming both physical (geographic and transportation) and scheduling (work commitments and wait times) barriers to specialty care for type 2 diabetes. In addition, telemedicine may enhance communication around diabetes care by improving information sharing between patients and clinicians. However, clinicians identify limited availability of home blood glucose data and vital signs as factors, which impair the optimal management of type 2 diabetes and related comorbid conditions via telemedicine. Previsit preparation, involvement of multidisciplinary providers, and frequent brief check-ins were identified by patients and clinicians as potential strategies to improve the quality of telemedicine care for adults with type 2 diabetes.

**Conclusions:**

Patients and clinicians identify key strengths of telemedicine in enhancing access to diabetes specialty care for adults with type 2 diabetes and describe approaches to ensure that telemedicine delivers high-quality diabetes care to patients who rely on it.

## Introduction

The use of telemedicine—synchronous, audiovisual, internet-based communication between patients and clinicians—to provide endocrinology care expanded rapidly in 2020 [1,2]. While many patients have since resumed in-person care, a significant proportion of patients continue to use telemedicine: more than 11% of endocrinology visits in a national sample from January 2022 to March 2023 were conducted via telemedicine [3]. Telemedicine can expand access to endocrinology care for patients in rural areas of the United States, where there are long-standing shortages of endocrinologists [4], and for patients who face transportation, mobility, or other barriers to in-person care. The use of telemedicine to increase access to specialty diabetes care is supported by national guidelines, which also support the need for additional research assessing components of successful implementation of telemedicine programs [5,6]. Evidence from randomized trials of telemedicine interventions for type 2 diabetes (T2D) demonstrates that remote review of blood glucose by care teams [7,8]; active remote medication adjustment [8,9]; patient engagement between visits via phone, text message, or portals [9]; multidisciplinary team involvement in virtual care [8]; and remote diabetes self-management education and support services [10-12] are associated with the greatest hemoglobin A_1c_ improvement and may support diabetes care quality. However, evidence on the benefits and limitations of real-world telemedicine approaches to provide endocrinology care to adults with T2D outside of trial settings is limited.

Retrospective analyses of real-world telemedicine outcomes for adults with T2D in primary care settings have had mixed results, with some studies finding equivalent or superior glycemic outcomes to in-person care [13-16], while others demonstrate inferior care quality [15,17,18]. However, evidence suggests that patients using telemedicine alone to access endocrinology care for T2D may not experience the same glycemic improvements as patients using in-person care [19]. We previously completed a survey study of diabetes specialists on factors impacting the quality of diabetes care delivered via telemedicine, in which clinicians cited clinical complexity, as well as limited clinical resources to support telemedicine, as factors that reduce effectiveness [20]. However, clinician and patient perspectives on the benefits and limitations of current telemedicine care delivery and approaches to improve this care have not been explored. As a result, in this study, we aimed to gain a deeper understanding of the perspectives of both diabetes specialty clinicians and patients on specific benefits and limitations of current telemedicine approaches for T2D and ways to enhance telemedicine effectiveness for patients who rely on it.

## Methods

### Study Design

In this qualitative study, we used a qualitative description approach to data collection and analysis. Qualitative description research studies aim to understand the perspectives or worldviews of participants with the goal of finding actionable insight; qualitative description is a common theoretical orientation for qualitative studies in the health sciences [21]. This theoretical orientation informed our study design, from participant selection to the development of the interview guide and data analysis [21]. Our goals of analysis were to describe the content of the interviews from the perspectives of study participants, without abstracting to the level of social theory [22]. Semistructured qualitative interviews were conducted with diabetes specialty clinicians from endocrinology clinics across the United States and patients from a single academic endocrinology center. The study team included an adult endocrinologist, a primary care provider, a qualitative methodologist, and two qualitative research analysts (one with a Master of Arts degree and one with a Juris Doctor degree, both male). We report our results based on the COREQ (Consolidated Criteria for Reporting Qualitative Research) framework [23].

Interview guides were developed by the study endocrinologist, primary care provider, and qualitative methodologist, based on findings of a previous mixed methods survey study of endocrinology patient and clinician experiences with telemedicine, specifically synchronous audiovisual communication or “video visits” for T2D, and were not pilot-tested [20]. Guides addressed patient and clinician perspectives on the current use of telemedicine to deliver or receive care for T2D, the benefits and shortcomings of telemedicine, and approaches to improve the quality of telemedicine care.

### Recruitment

Diabetes specialty care clinicians were recruited via direct email outreach in June 2023. All 44 clinicians targeted for recruitment worked in adult endocrinology clinics. Patient participants were recruited from respondents to a previous survey study about telemedicine for T2D conducted between August 2022 and March 2023. All 24 patient participants contacted for recruitment were adults aged >18 years with T2D who had used telemedicine in the past year to access endocrinology care at 1 of 7 clinical sites associated with a single large academic medical center.

### Data Collection and Analysis

Interviews with clinicians were conducted between June and August 2023. Interviews with patients were conducted between June and July 2023. Semistructured interviews were conducted by two trained qualitative research analysts via a secure videoconferencing platform and lasted 45-60 minutes. Audio-only transcripts generated via videoconferencing software were reviewed and corrected using notes recorded by each analyst during interviews. Interviews continued until each interviewer determined, through a review of transcripts and notes, that thematic saturation had been reached [24]. Participants did not have previous relationships with interviewers and did not receive any information about interviewers during this study. No one was present at the interviews except for the participant and interviewer. Transcripts were not returned to participants and participants did not provide feedback on the findings.

Initial codebooks were inductively developed by experienced qualitative research specialists for each dataset based on the content of the interviews. In this process, researchers reviewed transcript data for both patient and clinician interviews, respectively, to identify key concepts within the raw data that could produce a system of codes for categorizing the data. These codebooks were then reviewed and approved by the qualitative methodologist. For both sets of interviews, two coders trained in the codebook co-coded the initial transcripts (3 patient and 4 clinician transcripts, respectively), then met to adjudicate their coding and refine the codebook based on any coding disagreements or discrepancies that arose. Finalized codebooks are included as Multimedia Appendix 1 (patient) and Multimedia Appendix 2 (provider). They then applied the codebook to the remaining transcripts and assessed intercoder reliability via kappa statistics provided by MAXQDA (VERBI Software) coding software. The overall κ score for the provider coding was 0.77, indicating “substantial” agreement, and the overall κ score for the patient coding was 0.92, indicating “near perfect” agreement [25]. All coding differences were adjudicated to full agreement. This finalized coding was used to assist in both conventional content [26] and thematic analysis [27] of the transcripts. Both conventional content analysis and thematic analysis rely on familiarization with and organization of the data through coding. Following coding, a systematic review of all text segments associated with particular codes can yield additional insight. Conventional content analysis was used to summarize and describe what participants said. Thematic analysis, following the steps described by Braun and Clark [27], was then used to identify overarching themes or recurring patterns within the data that might not be identified by the summarization of content alone in the conventional content analysis. Themes were then reviewed and refined to ensure they accurately represented the data in the original context.

### Ethical Considerations

This study was approved by the University of Pittsburgh Institutional Review Board (study number STUDY23030092). All participants provided verbal informed consent before the interview. Audio-only transcripts generated via videoconferencing software were reviewed and corrected using notes recorded by each analyst during interviews, with identifying details redacted. Interviews continued until each interviewer determined, through a review of transcripts and notes, that thematic saturation had been reached [24]. Participants did not have previous relationships with interviewers and did not receive any information about interviewers during this study. No one was present at the interviews except for the participant and interviewer. Transcripts were deidentified and were not returned to participants, and participants did not provide feedback on the findings. Interviews with clinicians were conducted between June and August 2023. Interviews with patients were conducted between June and July 2023. Semistructured interviews were conducted by two trained qualitative research analysts via a secure videoconferencing platform and lasted 45-60 minutes. Participants were compensated with a US $50 cash card.

## Results

### Participants

Diabetes specialty clinicians (n=15) who completed interviews practiced in 12 unique institutions across 8 states (California, Florida, Maryland, Massachusetts, New York, Pennsylvania, Oregon, and Texas). In total, 14 clinicians were endocrinologists, and 1 was a nurse practitioner; 14 practiced at academic medical centers, with 1 in private practice.

Patients (n=13) who completed interviews all received care within a single academic endocrinology division, including 7 clinics across both urban and rural counties, and reported duration of T2D from 3 to 20 years. There were 29 clinicians and 14 patients who did not respond to recruitment emails or phone calls or reported they did not have time to participate.

Many clinician and patient participants reported using telemedicine for the first time during the COVID-19 pandemic. Clinicians described attenuation in use over time with a declining perceived need for social distancing due to patient and institutional preferences. On the other hand, many patient participants described a desire to continue to use telemedicine due to convenience, although some reported a preference for returning to in-person care.

### Findings

We identified 4 major themes around patient and clinician perspectives on key benefits of telemedicine for specialty care of T2D, limitations of current telemedicine practice, and approaches to improve the quality of diabetes care delivered via telemedicine.

#### Theme 1: Telemedicine Enhances Access to Diabetes Specialty Care by Overcoming Multiple Barriers to In-Person Care

Clinicians and patients generally agreed that one major benefit of telemedicine is improved access to care. Many clinicians described increasing access to endocrinology care for patients who face barriers to traditional office visits as a main reason for ongoing use. Clinicians cited multiple types of barriers faced by patients that telemedicine can help overcome: long travel times for patients who live at a significant distance from the clinic, transportation availability, and cost of transportation. Additional barriers including scheduling conflicts between in-person visits and work, as well as childcare or eldercare commitments, were also mentioned. Clinicians also perceived telemedicine to be beneficial in specific situations that require increased visit frequency, such as diabetes in pregnancy. In addition, clinicians noted that telemedicine may make it easier for patients with mental health conditions, such as depression, to access care by reducing the burden of attending visits. Importantly, clinicians noted that these factors which reduce barriers to care resulted in significantly lower no-show rates for telemedicine visits (Textbox 1).

Many patients also reported that telemedicine increased access to diabetes specialty care and made that care more convenient. Patients reported that telemedicine allows them to overcome the lack of transportation, as well as avoid costs for parking and gas. For example, one patient stated:

The pros are…travel time, wait time, you know I’m not using gas, I’m not using a vehicle, I’m not traveling.

In addition, patients reported significant benefits in saving time, both in traveling to the clinic and waiting to see their clinician, with telemedicine compared to in-person care (Textbox 1).

**Select quotes for theme 1: telemedicine enhances access to diabetes care in many ways.**

**Clinician perspectives**

- They come in from two, three hours away, and in those cases we’ll do telemedicine, just so that they’re not having to drive back and forth like five hours.
- We’re not in a wealthy area: a lot of people are having transportation issues, having trouble affording gas, um, have other issues like childcare or elder care, or, you know, can’t get off from work, so it makes it difficult for them to come to in-person visits.
- Lot of times people cancelled because, for a variety of personal reasons, they can’t get into the clinic, and it takes so much time to get into clinic or it costs money. But with telemedicine, I had almost a zero no-show rate.

**Patient perspectives**

- I think is much easier, because sometimes you can do all this money spending to get there, and they say the same thing they say every time.
- It’s just more convenient. I got work and I don’t have to take, like, a whole day of work off I can just schedule, you know, my lunch break.
- Telemedicine works a lot for me, being that I don’t always have transportation to get to my appointments.

#### Theme 2: Telemedicine Can Facilitate Information Sharing in Diabetes Visits

Clinicians and patients generally agreed that telemedicine can allow for more information-sharing diabetes visits, but had differing views on the specific ways telemedicine was most helpful. Clinicians reported that the ability to have caregivers engaged in visits is one major way telemedicine enhances information sharing, especially with regard to self-management of diabetes including diet and medication regimen (Textbox 2).

Immediate access to medications in the home was cited as another benefit of telemedicine, especially for patients on complex medication regimens:

When they are at home, I’m actually able to tell them, why don’t you go show me what exactly you’re taking, show me the color of the pen…so I think that helps me from a standpoint that if they are on a very complex regimen, I have a better way of assessing.

On the other hand, many patients focused on improved communication with their clinicians via telemedicine:

I think the communication just has improved. I mean [my clinician] can focus on being prepared…for the visit, where we can spend more time just discussing my goals and where I’m at.

Screen sharing to review glucose trends and other data was also discussed as one benefit of telemedicine visits for diabetes care, which enhances communication and information sharing. In addition, patients described reduced stress associated with telemedicine compared to coming into the clinic, including feeling more at ease and avoiding the hassle associated with navigating health care facilities and procedures. This reduced stress further improved their rapport and communication with their clinician via telemedicine (Textbox 2).

**Select quotes for theme 2: telemedicine can facilitate information sharing in diabetes visits.**

**Clinician perspectives**

- Having the family member there, knowing that the family member will encourage the patient to do what we’ve discussed when they leave the visit is very helpful.
- I’ll be like, “Hey, can I speak to your spouse or your children? Can they get on the phone? We can go over the plan.” That saves me time because it happens synchronously during that same visit, I don’t have to call the family member after the patient has left the clinic to update about the plan.
- I exactly know what the patient is taking because they are able to show me the bottles.

**Patient perspectives**

- I’m able to talk with the doctors more; you know…talking, we can get a little more things discussed; she can pull things up and show ‘em to me. I guess you can do that in person too, but, you know, it’s just really just convenient.
- I was very comfortable talking to her about the things I needed to talk to her about…I like the telemedicine because it’s, you know, I’m not like getting judged.
- I just seem more relaxed on the phone…There’s no office, you know, office mumbo jumbo, you know, waiting…vital signs at all that, I just don’t like any of that.

#### Theme 3: Clinicians and Patients Perceive Different Limitations of Current Telemedicine in Supporting Successful Diabetes Management

Clinicians and patients differed in their perspectives on the limitations of current telemedicine approaches for diabetes management. Clinicians described multiple drawbacks of telemedicine, which limit their ability to help patients manage diabetes during routine visits. The lack of glucose data, both from glucometers or when continuous glucose monitor device data are not automatically shared, was commonly cited as a major limitation. In addition, clinicians discussed increased difficulty in delivering care through telemedicine for patients with limited English proficiency due to challenges using interpretation services. For example, one clinician stated:

If the interpreter can’t log on via the video platform, then I have to…call the patient via the telephone with interpreter…not as seamless as doing an interpreter visit in clinic.

Clinicians also noted that telemedicine may be less effective for medically complex patients due to the limited ability to obtain vital signs and conduct a physical examination to inform management of comorbid conditions, such as hypertension. In addition, clinicians described how it can be challenging to leverage multidisciplinary care resources, such as diabetes self-management education and support, with current telemedicine protocols compared to in-person office visits (Textbox 3). As these services are often available on a drop-in basis in clinics, current telemedicine approaches may limit the ability of clinicians to provide these resources in an unscheduled manner as needs arise during video visits.

**Select quotes for theme 3: clinicians and patients perceive different limitations of current telemedicine in successful diabetes management.**

**Clinician perspectives**

- Most of my patients…do not keep a separate glucose log outside of their glucometer, and so it’s really challenging to try and understand…if someone’s on any, you know, agent that has a potential for hypoglycemia…how can I titrate that safely in the absence of data?
- Hypertension management is trickier via telemedicine unless someone has a blood pressure cuff at home and is checking their blood pressure…so I would say I have very seldom made adjustments to antihypertensives in a telemedicine-only visit.
- A lot of type two diabetes management also focuses on lifestyle, right? Like it focuses on things like you know, their diet, what their regular lifestyle is, the level of activity, etc. So, many times if it’s over telemedicine, I can’t use the other services that we can offer in person in clinic right then and there when the patient is there.

**Patient perspectives**

- Really is no big difference. The same conversation we would have, in-office, face-to-face, will be the same conversation we would have in, you know, telecommunication.
- I really wish I could have, you know, had my blood work and my blood pressure and everything done.
- Not being able to… get my A1C in person… that’s probably one of the…only other hardships that I didn’t like about it.

On the other hand, many patients perceived that telemedicine overall delivered a very similar quality of care to in-person visits. For example, one patient stated:

The telecommunication visit was good for me…there was nothing that I really needed to see my physician with in-person, that I needed to go over her that I couldn’t go over with her on the phone.

However, some patients described the drawbacks of not receiving in-person diabetes care, including the inability to have a physical exam, vital signs, and lab work done in the office (Textbox 3).

#### Theme 4: Strategies to Enhance the Effectiveness of Telemedicine Diabetes Care in the Future

Clinicians and patients also had differing, but complementary, perspectives on approaches to improve the current delivery of diabetes care through telemedicine to better help patients successfully manage T2D. Clinicians described two main strategies. The first centered around preparation before telemedicine visits to ensure that all information that would routinely be available in office visits is similarly available to clinicians during telemedicine visits. This could include the collection of glucose data and home-measured vital signs, as well as addressing any potential technological barriers to the successful completion of the visit. The second main approach included the engagement of interdisciplinary team members during visits and ensuring postvisit follow-up. As one clinician stated when asked about the ideal telemedicine visit:

I would finish my visit and send patient back to the Zoom waiting room, and then the… CDE or nutrition will join that visit or a psychologist…and… a nurse…to kind of reiterate the instructions that or the plan that we discussed during the visit, and then schedule the follow-up, obviously. That's sort of the, the dream flow of the televisit.

However, clinicians reported that inadequate staffing is the major barrier that prevents the implementation of these strategies in current practice (Textbox 4). Finally, some clinicians also emphasized the importance of changing policies regarding reimbursement to the future of telemedicine for diabetes care; as one clinician put it, “[if reimbursement rates go down] it's a concern because then we won't be able to do it. And I think care will suffer.”

**Select quotes for theme 4: strategies identified to enhance diabetes care through telemedicine in future use.**

**Clinician perspectives**

- So before the visit, would have CGM download or glucometer data for like two weeks, an updated list of their medications, episodes of hypoglycemia—that’d be very helpful to have ahead of time—and if they did have vitals from home, so if they were checking their blood pressure or weight if they had that information ahead of time, and then actually checking your blood sugar at the visit if that was part of the protocol, you know, getting…labs they were due for ahead of visit, that would be fantastic.
- So, optimal before the visit, every single person has uploaded data to a cloud… every single person has had necessary labs in order, and everyone has ensured that they can log into the app and have good internet...After the visit, you know, I think in an ideal world is that there would be some system that can prompt patients, remind patients, and then also alert me if they have not completed the next steps.
- I mean, so much of telemedicine success is based on the previsit work that’s done, and that’s all, you know, non-provider based. So, staffing is the biggest challenge that most practices have with trying to ensure to do the previsit calls, confirmation calls, ensuring all this stuff is done…that’s the biggest barrier I think, in ensuring that practices are adequately staffed to support the in-person volume, plus do all of this [telemedicine] stuff.

**Patient perspectives**

- Well, I’d kind of like to have more education, you know, cause I’ve never seen the diabetes educator through a video visit, and I’d really like to get more education. I think that the education is key to diabetes, And the more you know about it, the better you can control it.
- Once a month check-ins or checkups… or…me being able to send my results to them, like once monthly..., like weight or… things like that.
- I mean, just if there were any, you know, specific follow-up items, that I needed, you know, to do…being, sent a reminder or whatever, electronically, or something along those lines.

While most patients felt that current telemedicine practices worked well for them, some identified additional support that could complement clinicians’ approaches above to improve telemedicine care. Some patients reported desiring more of an opportunity to access diabetes education and meet with interdisciplinary team members through video visits (Textbox 4). Others felt that using telemedicine to complete more frequent check-ins on their diabetes or offer reminders between visits could improve their diabetes management by helping them stay on track:

A 10-minute checkup maybe once a month, once every other month. ‘Hi…What are your numbers? What’s your glucose? How are you feeling?’…Especially for those who haven’t, you know, been consistent.

## Discussion

### Principal Findings

This study provides an updated assessment of clinician and patient perspectives on the current use of telemedicine to deliver endocrinology care to adults with T2D more than 3 years after initial widespread uptake in the United States. Our findings add to previous literature by gathering perspectives from patients and endocrinology clinicians practicing in diverse clinics across the country on optimal practices to address the limitations to effective routine clinical diabetes care via synchronous telemedicine. Clinicians emphasized the importance of access to home blood glucose data and discussed how telemedicine can make it difficult to manage common comorbid conditions due to a lack of vital signs or other home monitoring data. These findings align with previous studies in which clinicians report that telemedicine is appropriate for less complex conditions and patients [20,28,29]. As a result, clinicians identify previsit preparation, including the collection of home health data as a key to promoting successful diabetes telemedicine visits, which has also been underscored in previous literature describing telemedicine practices in the United States [30]. In addition, our findings align with existing evidence from other countries, including Australia and the United Kingdom, which supports the importance of multidisciplinary care and access to education in leveraging technology for diabetes care, as well as the benefits of synchronous video visits in improving access, reducing the patient’s burden of treatment, and improving clinician-patient communication [31,32]. Clinicians also identified the shortcomings of current telemedicine approaches in integrating allied professionals, including translators, diabetes care and education specialists, and nutritionists, into visits. Both clinicians and patients identified engagement of the multidisciplinary care team as one approach to ensure the delivery of high-quality care remotely, which may be especially important for patients who are clinically complex. Finally, patients also identified that enhanced follow-up after visits and the use of telemedicine for more frequent, brief check-up visits would improve the diabetes care they receive virtually.

In this study, patients generally reported satisfaction with the communication, information sharing, and overall care received through telemedicine. In addition, patients emphasized increased convenience and reduced costs associated with transportation as major benefits. These findings align with and add to previous literature in which patients with diabetes identify time and cost savings as benefits of telemedicine, while generally being satisfied with quality of care [29,33-35]. However, previous literature also underscores patient concerns about the lack of physical examination, vital signs, and in-office laboratory work potentially reducing the quality of diabetes care accessible through telemedicine, issues which were also identified in this study [29,34,35]. Our findings that patients report telemedicine is less stressful and potentially enhances communication around diabetes care contrasts with other studies of adults with T2D [34] and other chronic conditions [36] in the primary care setting, in which inferior communication and rapport building were noted. This may be due to an emphasis on the review of home glucose data and increased use of continuous glucose monitoring in the endocrinology setting relative to primary care, which has been emphasized in previous studies as one key component to successful telemedicine visits [29].

Both clinicians and patients describe how telemedicine enhances access to care by removing barriers to in-person visits, consistent with previous literature [28,37]. Clinicians in our study also emphasized that telemedicine results in lower no-show rates than in-person care, a finding seen in previous studies in both diabetes and primary care clinics [37-39]. Adults with T2D who have geographic or transportation barriers to accessing specialty diabetes care already experience worse care quality [40-42] and higher diabetes-related mortality [43-45]. Thus, ensuring that telemedicine delivers care that is at least as high-quality as in-person is crucial to promoting equitable access to care. Policies that preserve reimbursement for telemedicine care and promote improvement of care delivery through telemedicine will be critical to continuing access to diabetes specialty care for underresourced populations.

### Limitations

Strengths of this study include providing an updated assessment of the perspectives of patients and clinicians on the current use of telemedicine for diabetes care more than 3 years after initial use when many centers have refined their virtual care delivery process. Importantly, this study includes diabetes specialty clinicians from across the United States; while most practice in academic centers, diversity in geography, patient populations, and local telemedicine protocols enhances the generalizability of our findings. However, clinicians from private practice are underrepresented in our sample, so findings may not apply to this practice setting. Patient participants were drawn from a single academic endocrinology division, which includes a diversity of geographic areas. However, findings may not apply to patients who receive endocrinology care for T2D at centers with different telemedicine care protocols.

### Conclusions

In conclusion, clinicians and patients perceive the important benefits of telemedicine in increasing access to care, especially for patients who face barriers to in-person care. Given the ongoing shortage of endocrinologists and the prevalence of barriers to in-person endocrinology care, some patients will continue to rely on telemedicine indefinitely in order to access diabetes specialty care for T2D. Thus, it is crucial to use insight from patients and clinicians to inform approaches to improve the quality of care delivered via telemedicine care to reduce existing disparities in diabetes care and outcomes for these populations. Ensuring adequate data sharing through previsit preparation, increased visit frequency based on patient needs, and engaging interdisciplinary teams during and after telemedicine visits can leverage the benefits of virtual care to ensure telemedicine is at least as good as, or even superior to, in-person specialty diabetes care for patients who rely upon it.

## Appendix

Multimedia Appendix 1 Patient codebook.

Multimedia Appendix 2 Provider codebook.

Multimedia Appendix 3 Supplementary File 3: COREQ Checklist.

## Abbreviations

COREQ: Consolidated Criteria for Reporting Qualitative Research
T2D: type 2 diabetes
