# Supplementary material for: Patient and Clinician Perspectives on the Effectiveness of Current Telemedicine Approaches in Endocrinology Care for Type 2 Diabetes: Qualitative Study
Source: JMIR Diabetes. 2025 Mar 11;10:e60765. doi: 10.2196/60765 (PMC11937712; doi:10.2196/60765)
Supplement: Multimedia Appendix 1 [file diabetes_v10i1e60765_app1.pdf]

Code System			Memo
Code System			
	Living with diabetes		Participants talk about thier experience living with diabetes, code when no other codes apply
		How long	Participant talks about how long they have had diabetes and thier experince with having diabetes since diagnosis
		Day to day	Participants talk about what they do day to day to manage and take care of thier diabetes.
		Insulin	Participants talk about if they have used insulin in the past, if they use it currently, and how often
		Technology	Participants talk about if they usetechnology to manage thier diabetes, such as an insulin pump or a glucose monitor
		Meeting goals	Participants talk about whether or not they feel they are meeting thier diabetes treatment goals. Also how their doctors feel about whether or not they feel they are meeting thier diabetes goals
	Telemed experience		Participants talk about thier general experience with using telemed for diabetes care, use when no other codes apply
		General experience	Participants talk about thier general experiences wiht using telemed for thier diabetes care
		How often	Participants talk about how often they use telemedicine for their diabetes care.
		Start	Participants talk about why they started to use telemedicine for diabetes care
		Future	Participants talk about if they plan to use telemed in the fute for diabetes care
		Likes/positives	Participant shares positive experiences with telemed and shares positives about thier endocrinologist
	In person vs video		Participants talk about thier experiances meeting with thier diabetes provider over video versus in person or talk about if they still see thier doctor in-person or if its just over telemed. Code when no other code applies
		Compare	Participants compare thier experiances meeting with thier diabetes providers over video versus in person
		Better	Participants talk about if there are any ways they find that video visits are better/easier for them than in person visits, also if video visits make it easier in any way for them to achieve thier treatment goals
		Harder	Participants talk about if there are any ways that video visits are harder/worse for them than in person visits. Also if video visits make it harder fro them to meet thier treatment goals
	Last few video visits		Participants talk about thier last few video visits for thier diabetes care. Use when no other codes apply.
		Went well	Participants talk about what went well during thier last few video visits with thier provider
		Did not go well	Participants talk about if there was anyhting that did not go well during the last few video visits with theit diabetes provider and if there is anyhting they would like improved

	Decision	Who made the decision for the visit to be done over video/telemed and why
Differnt aspects of care		Participants talk about thier experience of different aspects of care during video visits and how how the visit being video rather than in-person impacted these aspects of care. Use when no other codes apply
	Home glucose readings	Participants talk about thier experince with sharing thier home glucose readings with thier providers during video visits and if they've had any challenges
	Other measurements	Participants talk about sharing other measurements, such as weight and blood pressure, with thier provider during video visits
	Meeting with care team members	Participants talks about if they meet with other diabetes care team members, such as educators and nutritionists,
	Family members	Participants talk about if they involve family members or of they are a family member that helps with diabetes care and if experience any challenges during video visits
Communicating with provider		Participants talk about how easy or hard it is to talk with thier provider during and after video visits and how well they are able to understand thier provider. Use when no other codes apply
	Changes during visits	Participants talk about how well they would understand changes thier endo recomended during a video visit. This includes changes to medicaiotn dose and diet.
	Problems during visits	Participants explains if they talk about any problems they have with managing thier diabetes during video visits with thier provider. Such as med side effects, difficulty taking meds, etc
	Follow up tasks	Participants talk about if they had any follow-up tasks, such as scheduling referrals to other doctors, getting blood work, or starting new medicines and if the visit being done over video versus in person had any affect on how hard it was to complete these tasks
	Problems inbetween visits	Participants talk about how they contact thier provider if problems arise inbetween video visits
Sharing Info		Participants talk about what thier experince has been with thier PCP and endocrinologist communicating about thier care and how this has impacted thier care
Specific tests		Participants are asked about specific testing and other care that patients with diabetes are recommended to have annually, and how diabetes visits being done over telemedicine may impact this care, versus in person visits. Use when no other codes apply
	Foot examinations	Participants are asked if their provider ever examined ther feet over a video visit.
	Recomended lab work	Participants are asked what thier experience has been with your diabetes provider having access to their lab results. Ex. hemoglobin A1C, cholesterol, urine protien

	Eye exams	Participants are asked what their experiances have been with thier providers having access to thier eye exams
	Other conditions	Participants are asked about management of other conditions related to diabetes that your diabetes provider might also address, such as high blood pressure or high cholesterol.
	Immunizations	Participants are asked about immunizations, such as annual flu shots and the pneumonia vaccines, which are recommended for people with diabetes.
Telemed Changes		Participants talk about how telemedicine for diabetes care could be changed so that they can best reach their diabetes goals. Use when no other codes apply.
	Telemed to meet goals	Participants are asked to think about a time when you were not meeting your diabetes treatment goals, such as when your blood sugar was higher than your goal, or if you were not able to take your medicines or manage your diet and exercise the way you felt you should. Are there any ways that your diabetes care through telemedicine could have been improved to help you during those times?
	Steps to reach goals	Participants are asked - If you could wave a magic wand and create a series of steps your diabetes clinics and provider would follow to help you take the best care of your diabetes through telemedicine, what would these steps include?
	Before video visits	Participants are asked if there are any steps they would like thier providers to take beofre video visitis so that they can get the best care possible
	During video visits	Participants are asked if there are any steps they would like thier providers to take during the video visitis so that they can get the best care possible
	After video visit	Participants are asked if there are any steps they would like thier providers to take after their video visitis so that they can get the best care possible.
	Inbetween video visits	Participants are asked if there are any steps they would like thier providers to take inbetween thier video visitis so that they can get the best care possible
Anything else		Participants share any other thoughts they have about using telemed for thier diabetes care
