# Supplementary material for: Patient and Clinician Perspectives on the Effectiveness of Current Telemedicine Approaches in Endocrinology Care for Type 2 Diabetes: Qualitative Study
Source: JMIR Diabetes. 2025 Mar 11;10:e60765. doi: 10.2196/60765 (PMC11937712; doi:10.2196/60765)
Supplement: Multimedia Appendix 2 [file diabetes_v10i1e60765_app2.pdf]

Code System			Memo
Code System			
	Clinic/practice		
		Clinic workflow/support for telemed	Code for provider's answer to question on their clinic's support for ongoing telemed use. Also code whenever provider describes how telemed fits into their normal workflow in terms of weekly/monthly, in-patient/out-patient; e.g., "... in-patient, seeing patients twice a week, half of which are telemed; out-patient, I utilize telemed X...."
		Covid-19	Code whenever it's mentioned.
		Diabetes education/educator	Code when provider says they employ a diabetes educator/coach in their treatment regimen; code also for provider describing the importance of diabetes education for patients reaching treatment goals.
		Dietician/nutrition education/cooking classes	Code when provider mentions it.
		Exercise/other lifestyle	Code when provider mentions the importance of exercise and/or other lifestyle changes. Provider may discuss utilizing an exercise/lifestyle coach or trainer.
		Instruction in tech device +/or data sharing	Code when provider describes instructing patients on device usage, how to share/link data, etc.; provider may elaborate on doing this in-person vs. via telemed.
		Insufficient staffing/time	Code when provider describes their clinic lacking adequate staffing and/or time to support optimal T2D care; might mention in context of pre/post visit check-ins.
		No-show rate	Code when provider cites no-show rate; e.g., "My no-show rate decreased with use of telemed."
		Nurse, APP, PA,	Code when provider mentions Nurse, RN, LPN, NP; APP; PA.
		Therapist	Code when provider mentions Therapist/mental health provider .
		Optimal protocol description	Code when provider describes elements for their optimal protocol. Co-code Pre-visit/Visit/Post-visit/btwn. visits and otherwise as needed.
		Other specialists	Code when provider mentions other specialist; e.g., nephrologist, cardiologist, opthalmologist, podiatrist, etc.
		Past telemed use	Code for provider describing past telemed use for T2D, if different from current use. E.g., "We started telemed during Covid-19 and relied on it extensively during the pandemic...."
		PCP	Code whenever provider mentions PCP, including care coordination with. May mention relying on physical exams, labs, immunizations from PCPs; may cite chart review of such data in EMR. Co-code as needed. E.g., "Endocrinology practices are overwhelmed with patient load given insufficient staff and time, so there is a need to closely coordinate care with PCPs."
		Pharmacist	Code when provider mentions it.

Code System			Memo
		Post-visit/btwn. visits	Code when provider discusses post-visit or between visit treatment actions; may discuss how well patient is doing meeting care goals.
		Practice setting	Provider discusses where they work: academic medical center, private practice, etc.; other staff clinician variables; patient panel variables -- co-code as needed.
		Pre-visit	Code when provider describes pre-visit checklist activities: 'rooming' the patient day prior to visit; calling patient to ensure they're screened and prepared for the visit - co-code as needed.
		Preference for in-person visit	Code when provider expresses their own preference for an in-person visit in any context. Co-code as needed.
		In-person visit	Code when provider mentions something about in-person visits. Do not co-code with Preference for in-person visit.
		Reimbursement rate for telemed	Code when provider discusses it; e.g., may say telemed is unsustainable w/o reimbursement for telemedicine from insurance providers.
		Secondary language support (translators, etc.)	Code when provider mentions secondary language issues including need for/use of interpretors.
		Social/community services support; SW	Code when provider mentions social/community services and/or coordinator/worker thereof. Social workers.
		Specific steps for patients not meeting treatment goals	Code for provider describing steps specific for patients who have been unsuccessful in meeting treatment goals. Co-code as needed.
		Visit	Code only when provider discusses optimal protocol for telemed visit as distinct from pre/post-visit elements.
	Patient		
		Caregiver support	Code when provider describes level of caregiver support and/or how it informs greater or lesser telemed use; may describe in positive terms but code regardless.
		Childcare needs	Provider mentions patient issues with childcare; e.g., telemed useful for parents who can't access childcare.
		Comfort of home environment	Code when mentioned; e.g., provider may describe how patient comfort at home is a benefit of telemedicine.
		Elderly needs	Provider mentions elderly patients; co-code as needed; e.g., Tech capability of patient.
		Insurance profile: Medicare/Medicaid, etc.	Code when provider describes patient insurance profile.
		New vs. established patient	Code when provider describes new vs. established patients and their relative telemed use. Code also when telemed visit is contingent on a recent in-person vist; e.g., "Only patients whom I've seen in-person in the last X months will be seen via telemed." May co-code Clinic workflow/support for telemed.
		Patient engagement with care	Code when provider describes patient engagement and how it informs greater or lesser telemed use.
		Preference for in-person or telemed visit	Code when provider explicitly describes patient preference for a telemed OR in-person visit.

Code System		Memo
	Transportation/distance from clinic	Code when provider mentions patient transportation/travel factoring against in-person visits, making telemed more useful; might be in context of seasonal travel challenges, e.g., winter.
	Work – time off to attend visits	Code when provider mentions patient inability to take time off of work to attend in-person visits.
Data		
	No data	Code when provider describes what they do when lacking patient health data needed for a telemed visit. Co-code as needed.
	Diabetes treatment complexity	Provider comments on the scope of telemedicine for treating a complex diabetes treatment regimen.
	Social determinants of health	Code when provider mentions 'social determinants of health', including but not limited to, income, gender, race/ethnicity, education.
	Labs/tests	Bucket code for 'lab work' or 'tests' when specifics not mentioned.
	Medication list	Code when provider describes med lists/recs; may say patients are more able to share data via telemed.
	Blood glucose data	Code when provider discusses this; code only if no mention of CGM. Same as blood sugar.
	CGM	Code when provider discusses continuous glucose monitoring (& device).
	No CGM	Code when provider discusses T2D treatment when patient lacks CGM.
	A1C labs	Code when provider mentions A1C &/or hemoglobin.
	Cholesterol	Code when provider mentions cholesterol.
	Urine protein	Code when provider mentions urine protein.
	Obesity	Code when provider mentions obesity.
	Hyperlipidemia	Code when provider mentions hyperlipidemia.
	Physical comorbidities	Code when provider discusses physical comorbidities; do not code for hypertension (use Hypertension code).
	Mental comorbidities	Code when provider mentions mental health comorbidities; e.g., depression.
	Hypertension	Code when provider mentions hypertention and/or bp cuffs or other mechanism to gauge it; bp cuff to gauge bp locally at home (accessing recent bp readings from other offices: PCP, cardiology).
	Insulin pumps	Code when provider mentions it.
	Bluetooth connected pens; insulin cap	Code when provider mentions these devices; pen may not be bluetooth-connected.
	Weight/scales	Code when provider mentions weight, measuring weight, BMI, etc. Do not code for calorie/step counts (use that code).
	Calorie and/or step counts	Code when provider mentions calorie/step counts.
	Retinal/opthalmology	Code when provider mentions retinal/opthalmology screening or issues.

Code System			Memo
		Foot/other elements of physical examination, e.g., injection si	Code when provider mentions foot and other physical screening or issues, , e.g, injection sites.
		Immunizations	Code when provider mentions this.
	Tech platform		
		Clinic (or patient) tech platform(s)	Code when provider describes the tech platform used for telemedicine; LibreView // Dexcom Clarity for glucose data sharing // Epic EMR (e.g., Extended Care) // Epic Haiku // MyChart // WebX // Doximity // Zoom, etc. any app for telemedicine
		Tech capability of patient	Code when provider describes the tech capability of patients.
		Data uploading	Code when provider describes data uploading/sharing of data; could be cloud-based, or via other mechanism, e.g., via email.
		Phone: audio-only visit	Code when provider mentions audio-only phone visits.
		Lacking devices/technology	Code when provider mentions patients lacking devices/technology needed for telemed.
		Lacking wi-fi/cellular reception	Code when provider describes patients lacking wi-fi/cellular connectivity for telemed visits.
		Three-way telemedicine	Code when provider describes a telemed visit with a third party provider; e.g., diabetes educator, nurse, PCP, other specialist.
	Misc.		
		Easier	Code when provider says telemedicine makes something easier. Co-code as needed.
		Harder	Code when provider says telemedicine makes something harder. Code also when provider mentions anything as harder. Co-code as needed.
		No difference	Code when provider says there's no difference between telemedicine and in-person visits for a treatment element. Co-code as needed.
		Other thoughts/suggestions	Provider gives other thoughts and ideas about T2D telemedicine care or anything else; E.g., Centralized platform for uploading data from glucose meters, CGM devices, etc. into a cloud-based system for easy retrieval would make it easier for providers and patients; would benefit both telemed + in-person visits.
